# Metastasising ameloblastoma or ameloblastic carcinoma? A case report with mutation analyses

**DOI:** 10.1186/s12903-023-03259-6

**Published:** 2023-08-12

**Authors:** Pavel Hurník, Barbora Moldovan Putnová, Tereza Ševčíková, Eva Hrubá, Iveta Putnová, Josef Škarda, Martin Havel, Oldřich Res, Jakub Cvek, Marcela Buchtová, Jan Štembírek

**Affiliations:** 1 Laboratory of Molecular Morphogenesis, Institute of Animal Physiology and Genetics, Czech Academy of Sciences, Brno, Czech Republic; 2 Institute of Clinical and Molecular Pathology and Medical Genetics, Faculty Hospital and Medical Faculty Ostrava, Ostrava, Czech Republic; 3 Department of Histology and Embryology, Faculty of Medicine, Masaryk University, Brno, Czech Republic; 4 Department of Pathological Morphology and Parasitology, University of Veterinary Sciences, Brno, Czech Republic; 5 Department of Hematooncology, Faculty of Medicine, University of Ostrava, Ostrava, Czech Republic; 6 Department of Hematooncology, University Hospital Ostrava, Ostrava, Czech Republic; 7 Department of Anatomy, Histology and Embryology, University of Veterinary Sciences, Brno, Czech Republic; 8 Department of Clinical and Molecular Pathology, Faculty of Medicine and Dentistry, Palacky University and University Hospital in Olomouc, Olomouc, Czech Republic; 9 Department of Nuclear Medicine, University Hospital Ostrava, Ostrava, Czech Republic; 10 Department of Oral and Maxillofacial Surgery, University Hospital Ostrava, Ostrava, Czech Republic; 11 Department of Oncology, Faculty of Medicine and University Hospital Ostrava, Ostrava, Czech Republic

**Keywords:** Ameloblastoma, Carcinoma, Case report, Metastasizing, Malignant, Benign, Wnt pathway, BRAF, FANCA

## Abstract

**Background:**

Ameloblastic carcinoma and metastasising ameloblastoma are rare epithelial odontogenic tumours with aggressive features. Distinguishing between these two lesions is often clinically difficult but necessary to predict tumour behaviour or to plan future therapy. Here, we provide a brief review of the literature available on these two types of lesions and present a new case report of a young man with an ameloblastoma displaying metastatic features. We also use this case to illustrate the similarities and differences between these two types of tumours and the difficulties of their differential diagnosis.

**Case presentation:**

Our histopathological analyses uncovered a metastasising tumour with features of ameloblastic carcinoma, which developed from the ameloblastoma. We profiled the gene expression of Wnt pathway members in ameloblastoma sample of this patient, because multiple molecules of this pathway are involved in the establishing of cell polarity, cell migration or for epithelial–mesenchymal transition during tumour metastasis to evaluate features of tumor behaviour. Indeed, we found upregulation of several cell migration–related genes in our patient. Moreover, we uncovered somatic mutation BRAF p.V600E with known pathological role in cancerogenesis and germline heterozygous FANCA p.S858R mutation, whose interpretation in this context has not been discussed yet.

**Conclusions:**

In conclusion, we have uncovered a unique case of ameloblastic carcinoma associated with an alteration of Wnt signalling and the presence of *BRAF* mutation. Development of harmful state of our patient might be also supported by the germline mutation in one FANCA allele, however this has to be confirmed by further analyses.

**Supplementary Information:**

The online version contains supplementary material available at 10.1186/s12903-023-03259-6.

## Background

The diagnostic terms ‘malignant ameloblastoma’ and ‘ameloblastic carcinoma’ (AC) are both routinely used in clinical practice, although only the latter phrase is correct according to the current World Health Organization (WHO) classification [1]. A very similar nosological unit, metastasising ameloblastoma (MA), is often difficult to distinguish clinically from the aforementioned AC. While AC exhibits microscopic features of malignancy, regardless of the presence of metastasis, MA is a microscopically benign tumour, but it possesses the potential to metastasise [2, 3].

Metastasising ameloblastoma constitute only about 1%–4% of all ameloblastomas [4–6]. They commonly metastasise through the haematogenous route with the lungs being the most common metastatic site (75%–88%) [7]. These lung metastases can often remain clinically silent (i.e. fail to exhibit significant clinical symptoms); thus, they could remain undiagnosed for a long time [8]. The average age of diagnosis is 34 years, and men and women are affected equally. The median survival time from the diagnosis of MA is 17.6 years [9]. The mandible–maxilla ratio is 1.96:1 and the majority of lesions develop in the posterior region of the mandible without a predilection for either side of the jaws [1].

Ameloblastic carcinoma is a rare malignant neoplasm with a poor prognosis, accounting for approximately 2% of all odontogenic tumours [5, 10]. Still, it is the most common malignancy among odontogenic tumours arising from the odontogenic epithelium [11]. It has an aggressive clinical course with extensive local destruction [12]. AC may arise de novo (i.e. a lesion may develop directly from the remnants of the odontogenic epithelium in the jaws – primary type) from transformation of a long-standing primary benign lesion such as a odontogenic cyst [13], or from benign lesions that have been subjected to several surgical excisions (secondary type). The majority of AC arise de novo, and only a few cases of malignant transformations of pre-existing ameloblastomas have been described previously [14–16]. The clinical features of AC are similar to those of ameloblastoma [12]; the histopathological features include the lack of differentiation, hypercellularity, a high mitotic index, vascular invasion and neural invasion. Metastasis into cervical lymph nodes or other sites including vertebrae, pleura, the skull, the parotid glands, the diaphragm and the liver are typical for AC [17, 18]. Due to the limited understanding of this malignancy, no standard of care has been established yet; therefore, care largely depends on the clinical features of the specific patient and procedures in the particular hospital. Typically, surgical resection of removable metastases with wide margins is performed first, followed by radiotherapy, chemotherapy and/or biological treatment.

Here, we present a case of a young man with a recurrent and, later, metastasising mandibular lesion. We also use this case to illustrate the similarities and differences between these two types of tumours and the difficulty in distinguishing between them.

## Case presentation

### Clinical summary of the case with a focus on the histopathological features

**Fig. 1:**
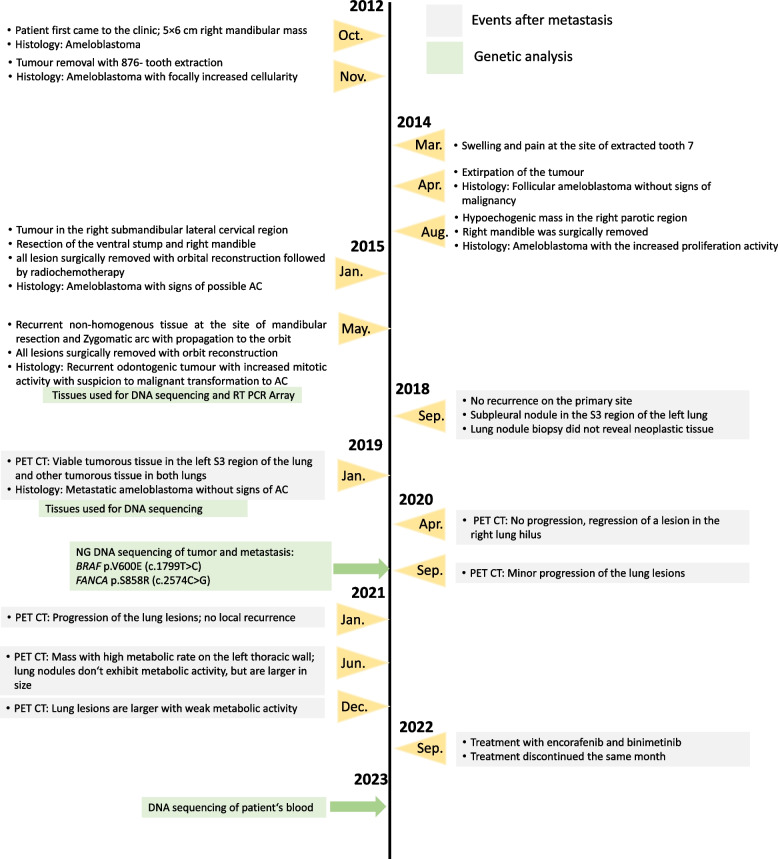
Timeline of diagnosis. Patient’s timeline with the important diagnostic events, treatment and collection of samples for sequencing between years 2012 to 2022

**Fig. 2:**
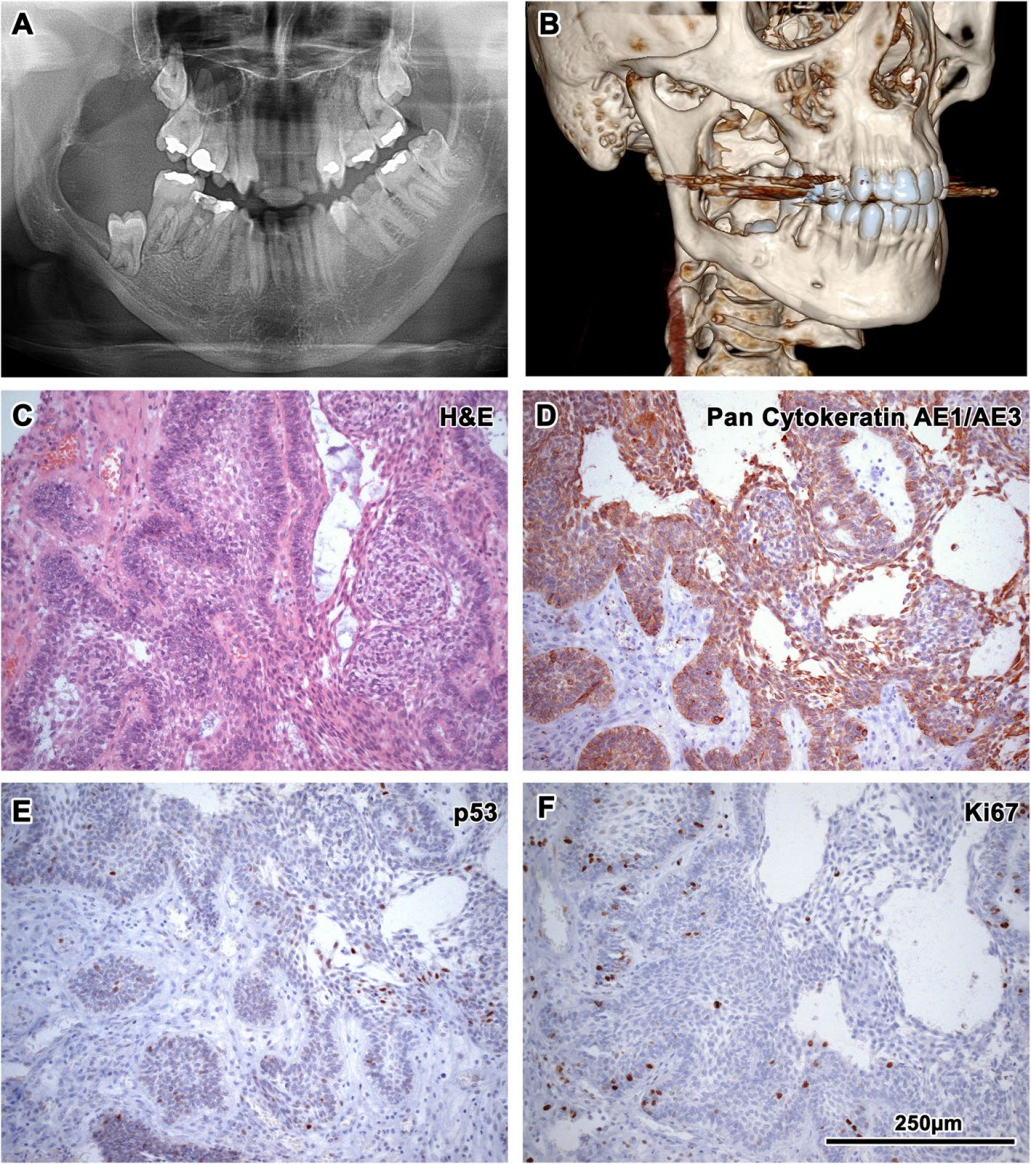
RTG and CT analysis and immunohistochemistry of the first collected oral sample of our patient. First X-ray of patient with ameloblastoma, where is retained wisdom tooth and loss of bone at the right side of mandible (**A**). 3D reconstruction of mandible from CT scans. There is a large lesion with bone resorption primary in the right side of the mandible extending into the region of the mandibular ramus (**B**). Epithelial cords of tumourous components with columnar cells exhibiting reverse polarity; Haematoxylin–Eosin staining, magnification 200x (**C**). Diffuse membranous positivity of pan-cytokeratin in tumourous cells; immunohistochemistry: CK AE1/AE3 (pan-cytokeratin (1:1, cat. no. 961, Abcam, UK), magnification 200x (**D**). Minimal nuclear immunoreactivity of p53 in tumour cells; immunohistochemistry: p53, magnification 200x (**E**). Slight nuclear positivity in Ki67 staining displays a weak proliferation activity of tumorous tissue; immunohistochemistry: Ki67 (1:200, cat. no. 275-R-16, Cell Marque, USA), magnification 200x (**F**). 3,3′-diaminobenzidine (DAB, cat. No. K3468, DAKO, Agilent Technologies, USA) was used to detect positive cells and Haematoxylin was applied to counterstain the nuclei

In October 2012, a patient was referred to our outpatient clinic by his dentist due to a slowly growing lump in the region of the lower right jaw, leading to right-sided swelling in the mandibular region (Fig. 1 - see for the timeline, Fig. 2). Orthopantomography detected a mass of approximately 5 × 6 cm with obvious bone erosion. The retained 8- tooth was encapsulated in the mass and located almost at the oblique line. An intraoral probatory excision was performed at the right retromolar area and histological diagnosis revealed ameloblastoma with typical reverse polarisation of tumour cells with subnuclear vacuolisation, no dentin and enamel formation and no significant cellular atypia. Computed tomography (CT) was performed, which detected a large (approximately 4.5 × 3.8 × 4.4 cm) well-delimited soft tissue swelling extending into the region of the mandibular ramus (Fig. 2). X-ray analyses of the lungs did not reveal any suspicious lesions. Subsequent tumour removal was combined with extractions of the retained tooth 8- and the irreparably damaged teeth 76–8.

In November 2012, the surgical extraction of 876- teeth combined with tumour removal from the right mandible was performed. The tumour was histologically verified as an ameloblastoma with focally increased cellularity and without other atypical features (Fig. 2).

In March 2014, new swelling and pain in the right mandibular region occurred. Orthopantomography displayed brightening at the site of the extracted tooth 7. In April 2014, a new extirpation of the tumour was performed, and the tumour was histologically verified as a follicular ameloblastoma without signs of malignity.

In August 2014, the patient exhibited another outgrowth of the tumorous tissue. Ultrasound of the right facial area and neck revealed a hypoechogenic mass in the right parotic region. Based on the clinical and X-ray findings, another surgery was performed and right half of mandible was removed. Histological analyses revealed recurrent ameloblastoma with foci of increased cellularity with elevated proliferative activity in Ki67 (> 20%) in these ‘hot-spots’. There was no tumorous tissue in parotic and submandibular lymph nodes.

In February 2015, the ultrasound examination revealed another tumour in the right submandibular lateral cervical region. Numerous reactive lymph nodes were present in the vicinity, one of which appeared possibly affected by metastasis (laterally from the tumour mass in the right lateral cervical region). A resection of the ventral stump of the right mandible was performed, together with a right-sided neck block dissection (I–III). The postoperative histological findings were recurrent ameloblastoma with signs of AC exhibiting a focal loss of organised basal cell stratification, stratum intermedium and stellate reticulum. Tumorous structures had expanded into the neighbouring lymph node. There were no histopathological features such as necrosis, pleomorphism, nuclear hyperchromasia or atypical mitosis.

**Fig. 3:**
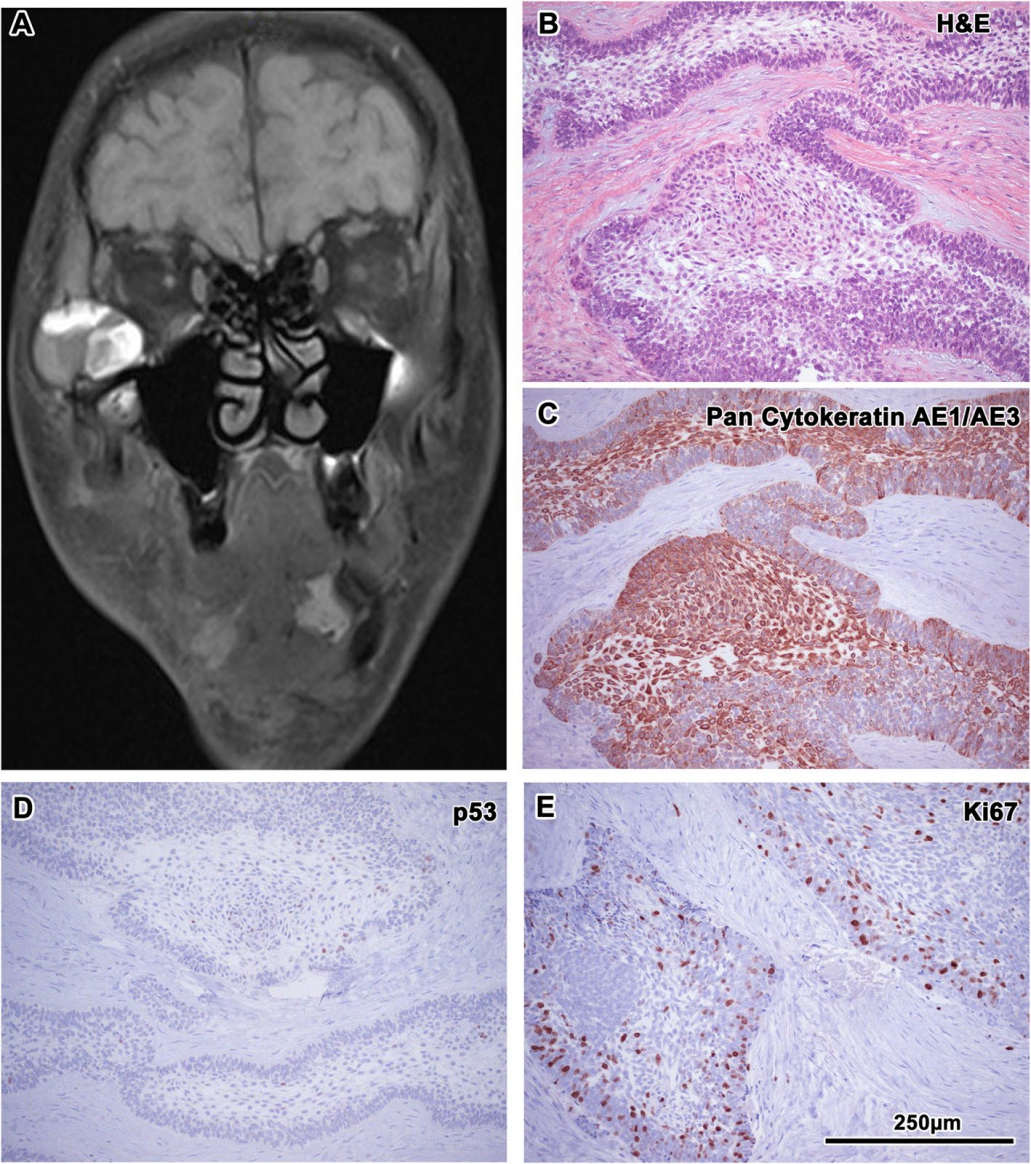
MRI and immunohistochemistry of the oral tissue sample after the tumor recurrence. MRI of head tumour recurrence into the region of the right zygomatic bone growing into the caudal part of the orbit (**A**). Epithelial cords of tumour compound of columnar cells with reverse polarization, dense cellularity in basal parts of tumour, increased mitotic activity and nuclear hyperchromasia; Haematoxylin–Eosin staining, magnification 200x (**B**). Diffuse membranous positivity of pan-cytokeratin with maximum of positivity in stellate reticulum-like cells in tumour tissue; immunohistochemistry: CK AE1/AE3 (pan-cytokeratin (1:1, cat. no. 961, Abcam, UK), magnification 200x (**C**). Weak nuclear immunoreactivity of p53 in tumour cell; immunohistochemistry: p53, magnification 200x (**D**). Nuclear positivity of Ki67 staining reaches the level up to 10% and represents increased proliferation activity; immunohistochemistry: Ki67 (1:200, cat. no. 275-R-16, Cell Marque, USA), magnification 200x (**E**). 3,3′-diaminobenzidine (DAB, cat. No. K3468, DAKO, Agilent Technologies, USA) was used to detect the positive cells and Haematoxylin was applied to counterstain the nuclei

In May 2015, a new swelling was observed in the right facial region. CT revealed a recurrent non-homogeneous tissue at the site of the mandibular resection and a new mass with erosions of the zygomatic arch. Magnetic resonance imaging detected ingrowth into the region of the right zygomatic bone propagating into the ventrocaudal part of the right orbit (Fig. 3). All these lesions were resected, and the right orbit was reconstructed with a titanium mesh. Histological examination verified the structures of a recurrent odontogenic tumour with foci of increased cellularity, increased mitotic activity and an increased nuclear–cytoplasmic ratio. These morphological features were evaluated as highly suspicious for possible malignant transformation into AC (Fig. 3).

**Fig. 4:**
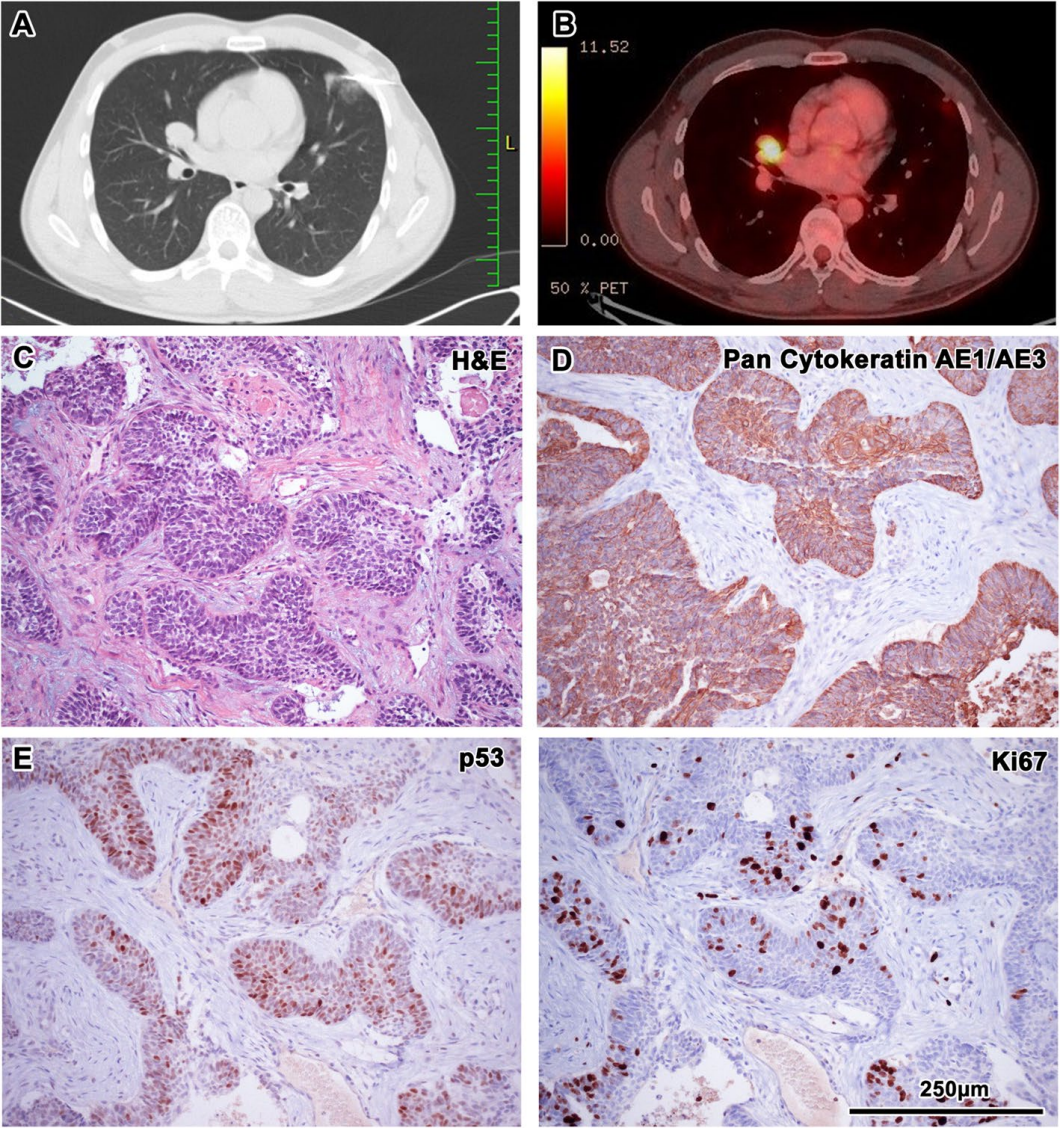
CT and PET CT analysis and immunohistochemistry of the lung metastases. CT of lungs with suspicious signs of tumour in the S3 region of the left lung (**A**). PET CT confirmed viable tumorous tissue in the S3 region of left lung (**B**). Epithelial cords located in dense fibrovascular stroma in lung tissue with features including high N/C ratio, increased mitotic figures. These features are suspicious for possible malignant behaviour of tumour, but not fully represent malignancy; Haematoxylin–Eosin staining, magnification 200x (**C**). Diffuse membranous positivity of pan-cytokeratin with the maximum of positivity in stellate reticulum-like cells of tumour. Immunohistochemistry—CK AE1/AE3 (pan-cytokeratin (1:1, cat. no. 961, Abcam, UK), magnification 200x (**D**). Nuclear staining of p53 in hot-spots; immunohistochemistry p53, magnification 200x (**E**). Nuclear positivity of Ki67 staining is increased up to 15–20% in cell-dense hot-spots and represents increased proliferation activity of the tissue; immunohistochemistry Ki67 (1:200, cat. no. 275-R-16, Cell Marque, USA), magnification 200x (**F**). 3,3′-diaminobenzidine (DAB, cat. No. K3468, DAKO, Agilent Technologies, USA) was used to detect the positive cells and Haematoxylin was applied to counterstain the nuclei

CT of the head and neck area taken in September 2018 did not reveal any signs of tumour recurrence. However, on the edge of the scan a 6 mm subpleural nodule was captured in the S3 region of the left lung, so CT of the lungs was performed; it confirmed a suspicious tumour (Fig. 4). A punch biopsy was performed under CT navigation in November 2018; it did not detect any invasive tumorous tissue. Nevertheless, subsequent positron emission tomography (PET) CT in January 2019 revealed viable tumorous tissue in the S3 region and other tumorous tissue in both lungs. After subsequent thoracoscopic resection of the S4 lobe, this tissue was histologically verified as a metastasis of ameloblastoma without clear micromorphological changes, promoting the diagnosis of AC (Fig. 4).

PET CT in April 2020 revealed no progression of the oncological disease; moreover, there had been regression of the lesion in the right hilum. In September 2020, new PET CT confirmed minor progression of the oligometastatic lesions in the lungs. PET CT in January 2021 detected the progression of multiple bilateral pulmonary lesions compared with the results from September 2020. Locally, in the area of the resected right mandible, there were no signs of disease recurrence. The next PET CT performed in June 2021 identified a non-specific mass of higher metabolic activity in the 3^rd^ intercostal region of the left thoracic wall. Multiple nodules of metastatic character in the lungs did not exhibit metabolic activity, but their size was morphologically larger compared with what had been observed in April 2021.

The last PET CT performed in December 2021 uncovered multiple nodulations in both lungs. They were relatively larger compared with the June 2021 examination and some of them displayed only a weak accumulation of fluorodeoxyglucose. There were also local post-therapeutic right-sided changes in the head and neck region without signs of recurrence.

Based on the data obtained from sequencing performed in February 2020 and revealed mutation in *BRAF* gene, the patient was administrated with encorafenib, small molecule BRAF inhibitor (300 mg/day) and binimetinib, inhibitor of BRAF-MEK signalling (60 mg/day). Three days after the drug administration, the treatment was discontinued due to the adverse reactions as impaired vision, itchy skin and pain in legs. In next week, the patient was administrated only to encorafenib (300 mg/day), however due to persistent skin itches this treatment was also terminated.

### Gene expression analyses revealed alterations in several members of the Wnt signalling pathway

**Fig. 5:**
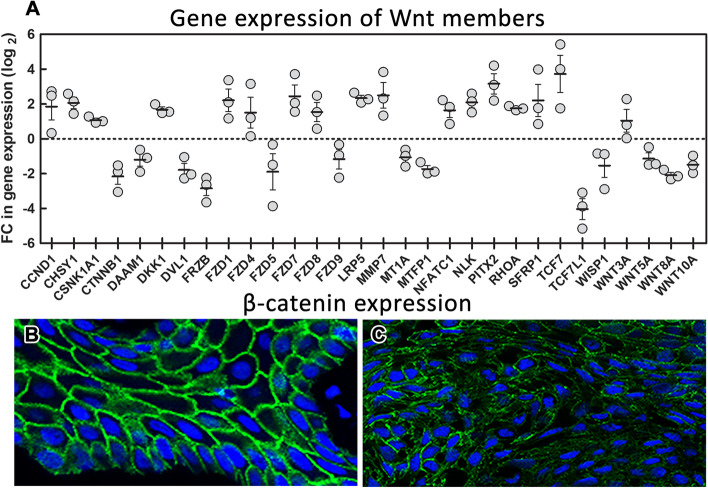
RNA expression analysis of Wnt related genes and β-catenin immunofluorescent detection. Expression analysis of genes related to Wnt signalling pathway. Ameloblastoma tumour tissue sample was compared to three healthy gingival tissues (represented by three data points for each gene). Values for gene expression changes were calculated using 2-^ΔΔCT^ method and subsequently log2 transformed (**A**). In the control oral epithelial cells, β-catenin signal is membranous, and it is regularly distributed through cell surfaces (**B**). In ameloblastic carcinoma, there is a signal loss in the tumorous cells and the expression pattern is discontinuous and stippled (**C**). Immunofluorescence: β-catenin (6B3) (beta-catenin (1:100, cat. no. 9582, Cell Signalling Technology, USA), the nuclei were counterstained by DRAQ5™ (cat. no. 62251, Thermo Fisher Scientific)

As the epithelial–mesenchymal transition (EMT) always plays a crucial part in metastatic epithelial neoplasms [19], we further followed possible changes in gene expression in tumorous tissue, which can be associated with our tumour behaviour. The Wnt pathway is one of the majors signalling pathways linked to EMT [20]. To uncover the possible aberrant gene expression of the major members of this pathway, we used a PCR array containing 84 candidate genes related to the Wnt signalling pathway. We analysed gene expression in the recurrent ameloblastic tumour tissue collected from the oral cavity (sample excised in 2015, Fig. 1) and compared it with gingiva from other healthy/non-tumorous patients used as controls (Fig. 5A).

Total RNA was isolated from the ameloblastoma and three samples of healthy gingiva (controls) using the RNeasy FFPE kit (cat. No. 73504, Qiagen, Germany) and transcribed into complementary DNA (cDNA) (gb Reverse Transcription Kit, cat. No. 3012, Generi Biotech, Czech Republic). The Human Wnt Signalling Pathway Plus PCR Array (cat. No. 330231 PAHS-043YA, Qiagen) was employed for gene expression analysis. The average Ct value of four selected housekeeping genes within the array panel was used for the normalisation. Relative gene expression for the ameloblastoma was calculated separately for each healthy gingiva as a fold change (FC) by the 2-^ΔΔCt^ method and log2 transformed. Thus, three individual values of gene expression were obtained for each gene within the array panel. Only data with the same trend of regulation for all three controls, with threshold cycle (Ct) values ≤ 34 for both control and test samples and those exceeding the average threshold [log2(FC) > 1 for upregulation, log2(FC) < -1 for downregulation) were considered relevant.

Our analysis indicated upregulation of the Wnt signalling pathway in the ameloblastic tumour potentially through the canonical signalling. One of the most upregulated genes was *PITX2*, identified previously as a transcriptional regulator of WNT3a in lung adenocarcinoma [21]. Another target whose transcription could have been activated by PITX2 is *CCND1* [22]. Detection of higher levels of growth-regulating CCND1 suggests increased proliferative potential of ameloblastic tissue.

The most upregulated and downregulated genes were the transcription factors *TCF7* and *TCF7L1*, respectively. Generally, these molecules have been suggested to play opposing roles. TCF7L1 is considered to be one of the repressors and TCF7 as one of the activators of Wnt target genes [23]. Moreover, we detected upregulation of Wnt receptors complex components, including several FZD receptors (*FZD1*, *FZD4*, *FZD7*, and *FZD8*), soluble frizzled-related protein (*SFRP1*) and the coreceptor *LRP5*.

We also found alterations in other genes apart from the core components that trigger and transduce Wnt pathway signals (Fig. 5A). Our analysis points to malignant transformation in the primary tumour through upregulation of the group of genes that have been previously related to migration and invasion in different types of tumours, including *RHOA*, *NLK*, *NFATC1*, *MMP7*, *DKK1* and *CHSY1*, and suppression of *CTNNB1*. As beta-catenin is important for cellular adhesions in epithelial tissues, we performed additional immunohistochemical analyses of tissue sections through tumorous tissue to evaluate possible changes in its expression on the protein level. We found only discontinuous CTNNB1 positivity of membranes or a punctuated pattern of its expression in tumorous epithelial cells (Fig. 5B, C) indicating disruption of cell–cell adhesions.

### DNA sequencing revealed a pathological BRAF mutation and a germinal FANCA mutation

To uncover the presence of potentially pathological mutations in our patient’s samples, we analysed DNA from primary tumorous tissue as well as from a lung metastasis by sequencing (Fig. 1) using a custom cancer gene panel including 93 genes of the highest importance for cancer biology (Table S1). The total DNA was isolated using the GeneJET FFPE DNA Purification Kit (cat. no. 73504, Thermo Fisher Scientific, USA) from the blood, primary tumorous tissue as well as from a lung metastasis. The library preparation was based on SureSelect platform (Agilent Technologies). The sequencing and analysis were outsourced to SeqMe s.r.o. (Czech Republic).

Raw data were automatically processed with the Basespace cloud interface (Illumina, USA) using default settings. Base calling, adapter clipping and quality filtering were carried out using bcl2fastq v2.20 Conversion Software (Illumina). Quality control of the samples was performed using the FastQC (v0.11.9) and MultiQC (v1.11) programs. Low quality bases (Q < 30) and adapter sequences were removed using TrimGalore (1.18).

Variant calling was performed using Agilent SureCall (v4.2.0.1) – All In One workflow using standard settings (identifying copy number variants [CNVs], single nucleotide polymorphisms [SNPs], indels and translocations; see below). The reads were aligned to the reference genome hg38 using BWA aligner. Variants were called by the Agilent SNPPET SNP caller algorithm. SNP filtering, mutation classification and annotation were applied to the list of called variants as a part of the analysis workflow. Variant annotation was performed based on various public sources, including NCBI, the Catalog of Somatic Mutations in Cancer (COSMIC), PubMed, Locus-Specific Databases and NCBI ClinVar.

We compared gene sequences of the tumorous tissue with known gene sequences in a public database (dbSNP). We found 50 variants (all non-synonymous) in the primary tumorous tissue and 133 variants in the metastatic tissue (of which 51 were non-synonymous), with a somatic variant in the androgen receptor (*AR*) gene being the only extra non-synonymous mutation in the metastatic tissue. There were found pathological mutations in the primary tumour and also in the patient’s lung metastasis samples in the genes *BRAF*, namely p.V600E (c.1799 T > C), and *FANCA*, namely p.S858R (c.2574C > G). The variant allele frequency was similar for primary tumour/metastasis in both cases (*BRAF* 28%/30%; *FANCA* 46%/46%), though the pathological interpretation of the *FANCA-*S858R mutation varies across the databases and publications, and it was confirmed to be pathological only when simultaneous deletion of second FANCA allele was present. The allele frequency of *FANCA-*S858R variant in public databases of human variation is ≤ 1%.

Moreover, we collected blood from patient to uncover possible germinal mutations in these genes (Fig. 1, Fig. S1). DNA was isolated using Blood and tissue kit (cat. no. 69504, Germany, Qiagen). Primers were selected to target above-described alleles in BRAF or FANCA (BRAF Fw CAGCATCTCAGGGCCAAAAAT, Rev GCTTGCTCTGATAGGAAAATGAG and FANCA Fw TCCGAAAGCTGCGTAAACCT, Rev CCATCCAGTTCGGAATGCAC). PCR was performed with Hot Start *Taq* DNA Polymerase (cat. no. M0495S, Bio Labs inc, USA) and PCR products were further processed with QIAquick PCR Purification Kit (cat. No. 28104, Germany, Qiagen) prior further sequencing, which was outsourced by Eurofins Genomics (Germany). Above-described mutations of BRAF for tumorous tissue were not found in patient blood and fibroblast cells cultivated from patients indicating somatic mutation in tumorous tissue and lung metastasis (Fig. S1). In FANCA, we uncovered in blood and cell cultures disease associated variant, c.2574C > G (p.Ser858Arg) similar to tumorous tissue indicating to represent germinal heterozygous mutation. These changes, however, were not found in tissues from two patients with cystic lesions in oral cavity that we used as controls (Fig. S1).

## Discussion and conclusions

### Comparison of histopathological features in ameloblastic carcinoma and metastasising ameloblastoma

Generally, it is difficult to differentiate benign ameloblastoma from MA based on histopathological evaluation alone as their histological appearance is almost identical, especially at early stages. The metastasising variant is, however, extremely rare; so far, only around 30 cases have been reported in the literature. Repeated surgical excisions of recurrent ameloblastoma have been suggested to support the development of AC [24], which could be the main cause of its appearance. In contrast, it is usually feasible to distinguish AC from the benign variants. It also represents a rare disease, with only a few hundred cases reported worldwide so far [25]. Features such as the presence of epithelial sheets, islands or epithelial trabeculae and the minimal presence or absence of stellate reticulum–like areas together with epithelial cells that display little or no differentiation towards columnar cells should alert the pathologist to the potential presence of malignant transformation. Other features that can justify the diagnosis of AC include cellular and/or nuclear pleomorphism, hyperchromatism, basilar hyperplasia, increased or abnormal mitotic activity, central keratinisation, necrosis and the presence of numerous cells with scant and clear cytoplasm [15, 26, 27]. Interestingly, our patient showed both forms, with different histological and clinical features and metastasising transformation that appeared over time.

### Wnt signalling in ameloblastic carcinoma and metastasising ameloblastoma

Alterations in the Wnt/β-catenin signalling pathway have been associated with more advanced/aggressive disease in several cancers [28]. β-catenin (encoded by *CTNNB1*) is a major part of intercellular connections, such as components of adherent junctions, and its aberrant expression has been linked to many malignancies and EMT [29]. In our patient, *CTNNB1* expression on RNA level was decreased in comparison to control tissue, and it was associated with weak β-catenin protein expression in membranes. Membranous expression of β-catenin has been described previously in AC [30, 31] as well as intranuclear expression [32], but the majority of ameloblastomas and AC exhibit loss of signal [30, 33]. Loosening of intercellular connections may have been associated with the progressive clinical course of the disease in our patient, eventually leading to EMT during metastasis. Mutations in *CTNNB1* [30, 34–36] and some silent mutations in *AXIN1* and *AXIN2* [37] have been described in ameloblastoma. So far, however, no mutations in these genes have been recorded in AC or MA, but not many such analyses have been up to now published. Moreover, these genes are not usually included in cancer panels.

Our patient had *PITX2* overexpression at the RNA level, which has been reported in one case of AC at the gene and protein level [38]. Another altered gene linked to Wnt signalling in ameloblastic tumours is *APC*. Cytoplasmic expression of APC negatively regulates canonical Wnt signalling [39] and its strong protein expression was recorded in two cases of MA [40], while mutation in *APC* was found in two cases of AC [32]. In our case, *APC* expression was upregulated at the RNA level. Alterations in Wnt signalling in our case probably implicate the initiation of metastatic transformation and cellular behavioural changes, such as the loss of cell adhesion, polarity modifications or increased migration potential, which we observed.

### Differences of the mutation profile in ameloblastic carcinoma and metastasising ameloblastoma

Our DNA sequencing revealed a pathological mutation in the *BRAF* proto-oncogene. BRAF-V600E is a gain-of function mutation that increases basal activity of the BRAF protein. The frequency of the BRAF p.V600E mutation is higher in ameloblastoma (64% in conventional, 81% in unicystic and 63% in peripheral) than in AC (35%) [41], where it is linked to aggressive behaviour of this tumour [42]. Moreover, mutations in *TP53* (encodes p53) [43] and *CDKN2A* (encodes p16) [44] have been reported in AC. In MA, mutations in genes such as *BRAF*, *MYCN*, *ARIDIA*, *MLL2*, *RUNX1* or *ASXL1* have been described [45]. Therefore, besides mutations in *BRAF*, different mutation profiles have been found in AC and MA, which could help with differential diagnosis between these two lesions. 

BRAF inhibition is also known to induce hyperactivation of Wnt signalling in several types of cancers such as colorectal carcinoma or melanoma, and Wnt pathway activation is responsible for the resistance of tumours to treatment using BRAF inhibitors [46]. Moreover, Wnt pathway members are frequently mutated in BRAF-mutant colorectal cancers, and co-mutation of *BRAF* and *APC* generates an extremely aggressive neoplastic phenotype associated with poor patient outcome [32, 47].

In tumorous tissue as well as in lung metastasis, we uncovered potentially pathological mutation in the tumour suppressor *FANCA* gene, which had not previously been associated with any type of ameloblastoma and since it was proven to be germline in this patient the effect is unclear. *FANCA* belongs to the Fanconi anaemia complementation group gene family. FANCA is involved in regulation of chromosome stability, and germline mutations in *FANCA* are mostly related to anaemia development (Fanconi anaemia) [48]. Our sample possesses the germline heterozygous S858R mutation that causes Fanconi anaemia when the second *FANCA* allele is mutated/deleted [49]. It negatively effects the ubiquitination function of the *FANCD2* gene. However, the predisposition of the heterozygous mutation S858R was not confirmed previously, when tested on pancreatic tumour or breast cancer samples, since this mutation was present in control as well as disease groups [50, 51]. On the other hand, because a main role of the FANCA protein is DNA damage repair, germline mutations in FANCA group have also been associated with higher occurrence of sporadic cancer [52–54]. Other *FANCA* heterozygous germline gene mutation along with several other gene mutations were found in patient with multiple primary malignancies (thyroid papillary carcinoma and gastric adenocarcinoma) [54]. The authors speculated that because FANCA is involved in mismatch repair, it can increase the mutational rate and lead to the development of multiple tumours. We propose that a similar effect could have caused the metastatic behaviour of ameloblastoma in our study [55]. To our knowledge, this is a first report of a mutation in this gene in a patient with ameloblastic tumour. Interestingly, previously it was also found that patients with Fanconi anaemia have a higher probability of developing oral cavity tumours [56].

Here, we have presented an interesting case of ameloblastoma transforming into AC and have provided a detailed report of its histopathological changes over time. We performed gene expression analyses to improve the understanding of this transformation and found that Wnt signalling is altered on several levels. These molecules might be future key targets for testing the ability to predict cancer behaviour and for their potential to be used as markers of early cellular changes during malignant transformation.

### Supplementary Information

**Additional file 1.**

**Additional file 2.**

**Additional file 3.**

## Data Availability

The datasets used and/or analysed during the current study are stored in the repository of the University Hospital Ostrava because they include private information about patient, but no-personal information are available from the corresponding author on reasonable request.

## Abbreviations

AC: Ameloblastic carcinoma
CNVs: Identifying copy number variants
CT: Computed tomography
EMT: Epithelial–mesenchymal transition
MA: Metastasising ameloblastoma
NCBI: National Center for Biotechnology Information
PCR: Polymerase chain reaction
PET: Positron emission tomography
SNPs: Single nucleotide polymorphisms
WHO: World Health Organization
