# Supplementary figures and images for: Metastasising ameloblastoma or ameloblastic carcinoma? A case report with mutation analyses
Source: BMC Oral Health. 2023 Aug 12;23:563. doi: 10.1186/s12903-023-03259-6 (PMC10423427; doi:10.1186/s12903-023-03259-6)

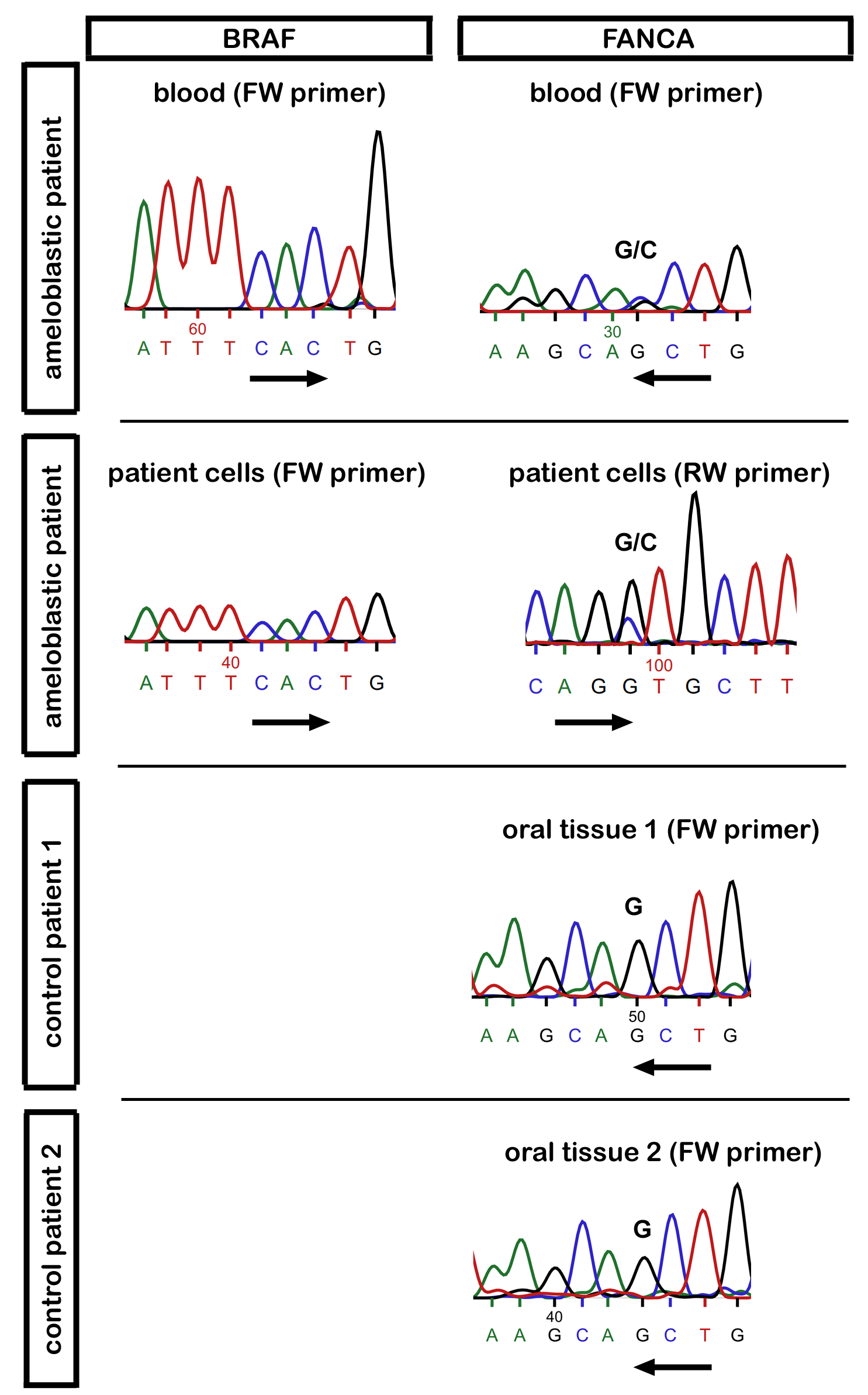

Supplement: Supplementary file 3 — Additional file 3. [file 12903_2023_3259_MOESM3_ESM.tif]
